# Targeting Nupr1‐Mediated p53 Mitochondrial Sequestration Alleviates Aortic Dissection by Restoring the Gadd45b‐Dependent Survival Program

**DOI:** 10.1002/advs.78143

**Published:** 2026-10-15

**Authors:** Xi Yang, Ke‐Yuan Chen, Yue Tian, Zong‐Sheng Jiang, Jian Lei, Xing‐Hui Zhuang, Jiang‐Bin Wu, Le‐Le Tang, Hao‐Yu Li, Xiao‐Hui Chen, Yue Wang, Yu‐Mei Li, Zhi‐Huang Qiu, Zi‐Wen Cai, Liang‐Wan Chen

**Affiliations:** ^1^ Department of Cardiovascular Surgery Fujian Medical University Union Hospital Fuzhou China; ^2^ Key Laboratory of Cardio‐Thoracic Surgery (Fujian Medical University) Fujian Province University Fuzhou China; ^3^ Engineering Research Center of Tissue and Organ Regeneration Fujian Province University Fuzhou China; ^4^ Department of Anesthesiology Union Hospital Fujian Medical University Fuzhou China; ^5^ The School of Pharmacy Fujian Medical University Fuzhou China; ^6^ Fujian Center for Safety Evaluation of New Drug Fujian Medical University Fuzhou China

**Keywords:** aortic dissection, apoptosis, mitochondrial dysfunction, Nupr1, VSMCs

## Abstract

Aortic dissection (AD) is a catastrophic cardiovascular emergency triggered by severe pathological stress. While continuous stress drives vascular smooth muscle cell (VSMC) apoptosis via mitochondrial dysfunction, the precise upstream orchestrators linking these stress signals to the mitochondrial apoptotic cascade remain elusive. By integrating clinical observations and single‐cell RNA sequencing, we found that Nuclear Protein 1 (Nupr1), a critical stress‐responsive factor, is significantly upregulated in aortas from AD patients and murine models, with enrichment in VSMCs. Remarkably, VSMC‐specific Nupr1 deficiency significantly ameliorated AD progression in vivo. Mechanistically, Nupr1 undergoes massive mitochondrial accumulation in Ang II‐induced apoptotic VSMCs. Rather than operating as a conventional transcriptional regulator, mitochondrially enriched Nupr1 functions as a pathological spatial switch. It sequesters p53 within mitochondria, driving transcription‐independent apoptosis while simultaneously blunting the protective Gadd45b‐mediated survival program. Furthermore, in vivo Gadd45b overexpression was sufficient to rescue mitochondrial dysfunction and ameliorate vascular collapse. Taken together, our findings conceptualize the Nupr1‐p53‐Gadd45b axis as an important determinant of VSMC fate and provide a novel and promising therapeutic strategy for preventing AD progression.

## Graphical abstract

This study identifies Nupr1 as a crucial stress‐responsive orchestrator driving AD. Under severe stress, Nupr1 massively accumulates within mitochondria to sequester p53, triggering apoptosis and concurrently repressing the *Gadd45b‐mediated* survival program. Targeted genetic interventions disrupting this pathological interaction restore mitochondrial homeostasis and prevent vascular collapse, offering a promising therapeutic strategy.

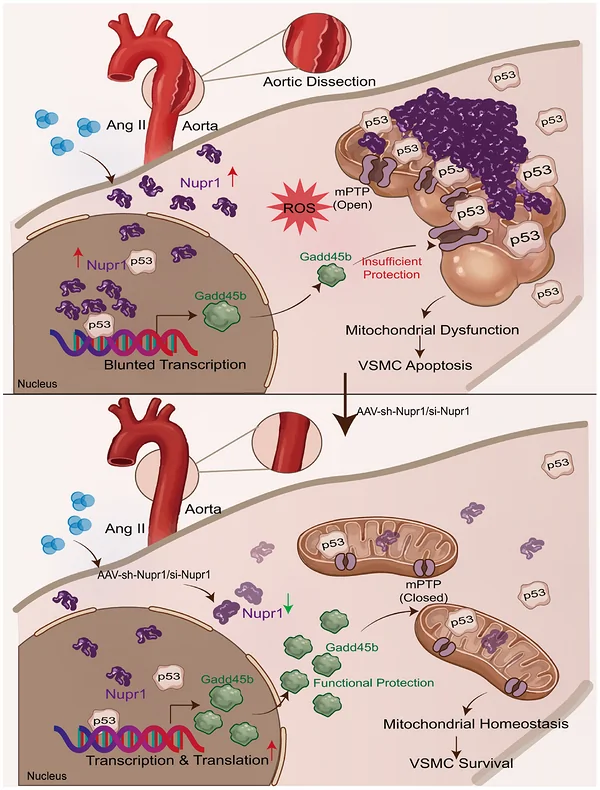

## 1 Introduction

Aortic dissection (AD) is a catastrophic vascular disease characterized by rapid progression and high mortality [1, 2]. Despite advances in surgical and endovascular management, no established pharmacological therapy is currently available to prevent AD progression. Therefore, a deeper understanding of the molecular mechanisms of AD is essential for the development of effective therapeutic strategies.

Vascular smooth muscle cells (VSMCs) are indispensable for maintaining vascular structural integrity. Progressive loss of VSMCs through apoptosis is a prominent pathological feature of AD and contributes to medial degeneration, aortic wall weakening, and eventual dissection or rupture [3]. Among the multiple mechanisms implicated in aortic wall remodeling, apoptosis of VSMCs driven by mitochondrial dysfunction has emerged as an important contributor [4]. It is well established that impaired mitochondrial bioenergetics and excessive opening of the mitochondrial permeability transition pore (mPTP) compromise mitochondrial integrity and promote cell apoptosis [5]. However, the key upstream stress‐responsive regulators that link mitochondrial dysfunction to VSMC apoptosis during AD pathogenesis are still elusive.

In the search for such critical upstream regulators, Nuclear Protein 1 (Nupr1) represents a compelling candidate. Also known as p8, Nupr1 is a transcriptional co‐regulator implicated in cell fate decisions, particularly in the regulation of apoptosis and autophagy [6, 7, 8]. As an intrinsically disordered protein [9], Nupr1 functions through dynamic interactions with multiple molecular partners, thereby coordinating context‐dependent signaling networks [10, 11]. Beyond its established roles in cancer biology, emerging evidence indicates that Nupr1 acts as a significant modulator in cardiovascular diseases, including pulmonary hypertension and cardiotoxicity [10, 12, 13]. These observations suggest that Nupr1 may act as an important mediator of stress‐induced vascular injury. However, its specific pathophysiological role and the underlying mechanisms within the vascular wall during the progression of AD remain completely unknown.

Accordingly, we sought to determine whether Nupr1 promotes AD progression and to explore the underlying mechanisms. Here, we demonstrate that Nupr1 deficiency in VSMCs mitigates mitochondrial dysfunction, suppresses apoptosis, and ameliorates AD progression. Mechanistically, our data reveal that stress stimulation triggers a massive accumulation of Nupr1 within the mitochondria. Independent of its canonical role as a transcriptional co‐regulator, this mitochondrial‐enriched Nupr1 is strictly required for the pathological sequestration of p53 within mitochondria. This specific spatial interaction not only provokes transcription‐independent mitochondrial apoptosis but also concurrently represses the protective Gadd45b‐mediated survival program. Ultimately, our findings identify the Nupr1‐p53‐Gadd45b axis as a critical driver of VSMC fate, providing a novel therapeutic target for preventing AD.

## 2 Results

### 2.1 Nupr1 Expression Was Increased in Patients With AD and Mice With AD

Compared to aortic tissues of non‐AD patients, the aortic media from AD samples exhibited severe disorganization and a significant loss of VSMCs, as evidenced by reduced nuclear density (Figure 1A,B). Given that this progressive cellular loss acts as a fundamental driver of aortic wall destabilization [14], we sought to uncover the underlying molecular orchestrators. To identify candidate genes potentially related to vascular changes in AD, a single‐cell RNA sequencing dataset GSE222318 was obtained from the Gene Expression Omnibus database [15, 16]. Uniform manifold approximation and projection (UMAP) analysis revealed 11 major transcriptionally distinct cell clusters (Figure 1C). VSMC clusters were specifically identified by the high expression of markers such as *ACTA2*, *MYH11*, COL8A1, *CNN1*, *TAGLN2, and DCN* (Figure S1A–F). Given the pivotal role of VSMCs in AD pathogenesis [17], we focused our subsequent analysis on this cell population. Chord diagram analysis of differentially expressed genes, centered on critical cell fate pathways (apoptosis, inflammation, cell cycle, and differentiation), revealed Nupr1 as a highly connected node, exhibiting pronounced expression changes across these interrelated processes (Figure 1D). Furthermore, although Nupr1 expression was elevated in both synthetic SMCs and macrophages within AD samples, its most prominent upregulation was localized to the synthetic SMC cluster (Figure 1E). To validate these sequencing findings, we measured Nupr1 expression in human and murine AD tissues. Real‐time quantitative polymerase chain reaction (RT‐qPCR) and Western blot analysis confirmed that Nupr1 protein and mRNA levels were significantly increased in both human (Figure 1F,H,I) and murine AD samples (Figure 1G,J,K). Subsequent immunofluorescence co‐staining revealed that although Nupr1 was elevated in both endothelial cells (Figure S2A,C) and macrophages (Figure S2B,D) of human aortic tissues, its accumulation in aortic VSMCs was uniquely accompanied by a drastic reduction in the contractile marker α‐SMA in both human (Figure 1L and Figure S1G) and murine (Figure 1M and Figure S1H) tissues. Given that α‐SMA deficiency reflects severe VSMC structural impairment, which is widely recognized as a fundamental driver of AD [18], we focused our subsequent mechanistic investigations specifically on the pathogenic role of Nupr1 within the VSMC population.

**FIGURE 1:**
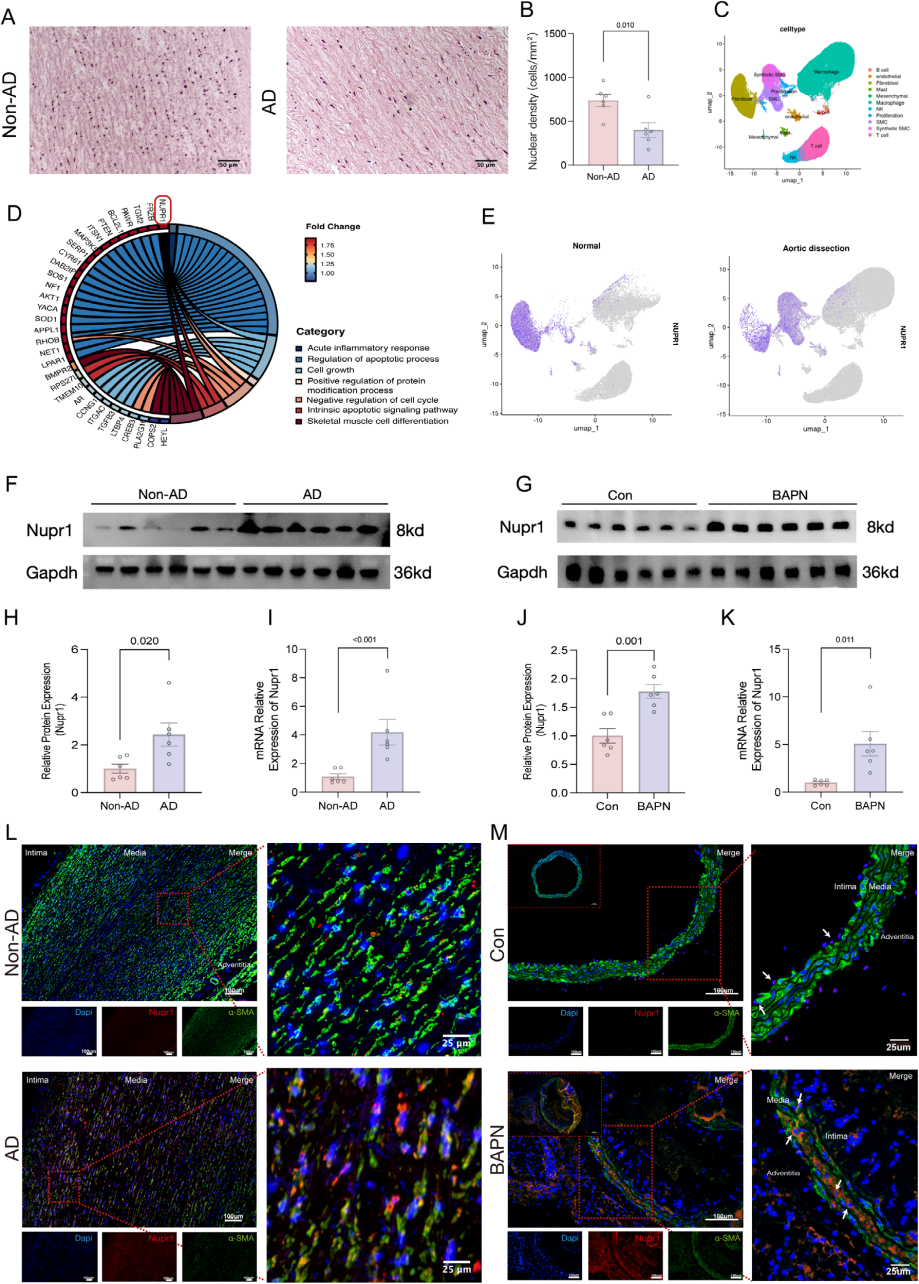
Nupr1 Expression Was Increased in Patients with AD and Mice with AD. (A) Representative macroscopic images of aorta cross‐sections stained with H&E staining. (B) Quantification of nuclei density (cells/mm^2^) in the aortic media from non‐AD and AD clinical samples (*n* = 6 per group). (C) Uniform manifold approximation and projection (UMAP) plot of 11 main cell types from aortic tissues. (D) Chordal diagram of GO pathways related cell‐fate and the contributing DEPs. The left side shows DEPs, and the color indicates the degree of difference. (E) UMAP analysis displaying Nupr1 expression in the different clusters between normal and AD groups. (F) Expressions of Nupr1 in aortic tissues from non‐AD and AD patients measured by Western blot. (G) Expressions of Nupr1 in aortic tissues from Con and BAPN‐induced AD mice measured by Western blot. (H) Quantification of Nupr1 expression in aortic tissues from patients with AD and non‐AD measured by Western blot (*n* = 6 per group). (I) RT‐qPCR analysis of Nupr1 mRNA levels in aortic dissection from patients with AD and non‐AD. Data were presented as relative fold change to controls (*n* = 6 per group). (J) Quantification of Nupr1 expression in aortic tissues from Con and BAPN‐induced AD mice measured by Western blot (*n* = 6 per group). (K) RT‐qPCR analysis of Nupr1 mRNA levels in aortic dissection from Con and BAPN‐induced AD mice. Data were presented as relative fold change to controls (*n* = 6 per group). (L) Representative images of Nupr1 expression using immunofluorescence staining in patients with non‐AD and AD aortic sections, and counterstaining with α‐SMA and DAPI. (M) Representative images and quantification of Nupr1 expression using immunofluorescence staining in mouse aortic sections and counterstaining with α‐SMA and DAPI.

### 2.2 VSMC‐Specific Nupr1 Deficiency Ameliorates AD Progression and Mitochondrial Apoptosis In Vivo

To elucidate the role of Nupr1 in AD development, we used BAPN‐induced AD mouse model for in vivo study. Four‐week‐old male mice (*n* = 30 each group) were fed with BAPN in drinking water and subjected to subcutaneous Ang II using osmotic minipumps to construct an AD model. The mice were intravenously injected with AAV‐sh‐Nupr1 or control vectors to assess its role in AD pathogenesis (Figure 2A). Nonabnormal aortic morphology was observed in mice from the saline group, while AAV‐sh‐Nupr1 administration in mice significantly relieved the severity of AD compared to that in BAPN+Ang II and BAPN+Ang II+AAV‐NC groups (Figure 2B). 60% (18 of 30) of BAPN+Ang II‐treated mice and 66.7% (20 of 30) of BAPN+Ang II+AAV‐NC‐treated mice died from aortic dissection and rupture. Notably, the mortality was attenuated in mice with BAPN+Ang II+AAV‐sh‐Nupr1 mice (36.7%, 11 of 30) (Figure 2C). In addition, the incidence of AD in BAPN+Ang II+AAV‐sh‐Nupr1 mice (56.6%, 17 of 30) was significantly lower than that in BAPN+Ang II+AAV‐NC mice (90%, 27 of 30) (Figure 2D). Moreover, systolic blood pressure was comparable between the BAPN+Ang II+AAV‐NC and BAPN+Ang II+AAV‐sh‐Nupr1 groups (Figure S3A,B), suggesting that the protective effect of Nupr1 knockdown is largely independent of systemic blood pressure. The transduction efficiency of AAV‐sh‐Nupr1 was validated by Western blot (Figure 2E–G), fluorescence staining (Figure S3C), and ELISA (Figure S3D). Importantly, co‐immunofluorescence staining with cell markers confirmed that AAV‐mediated knockdown was highly specific to VSMCs, with no significant reductions observed in nontarget cell populations, including endothelial cells, adventitial fibroblasts, or macrophages (Figure S4A,B). Hematoxylin and eosin (H&E) and Elastin Van Gieson (EVG) staining revealed that AAV‐sh‐Nupr1 mitigated BAPN+Ang II‐induced vascular pathology and disruption in elastin alignment (Figure 2H,I and Figure S3E). TUNEL staining revealed that AAV‐sh‐Nupr1 alleviated BAPN+Ang II+AAV‐NC‐induced VSMCs apoptosis in the media (Figure 2J,K). Subsequently, transmission electron microscopy (TEM) showed that AAV‐sh‐Nupr1 treatment alleviated the abnormal appearance of mitochondria in aorta sections, such as swelling and impairment of mitochondrial double‐membrane integrity, which was caused by BAPN+Ang II‐treatment (Figure 2L). Treatment with AAV‐sh‐Nupr1 decreased mitochondrial size and circularity index, compared to that in BAPN+Ang II+AAV‐NC mice (Figure 2M). Together, these data indicate that deficiency of Nupr1 ameliorated AD pathogenesis, dysfunctional mitochondria, and apoptosis.

**FIGURE 2:**
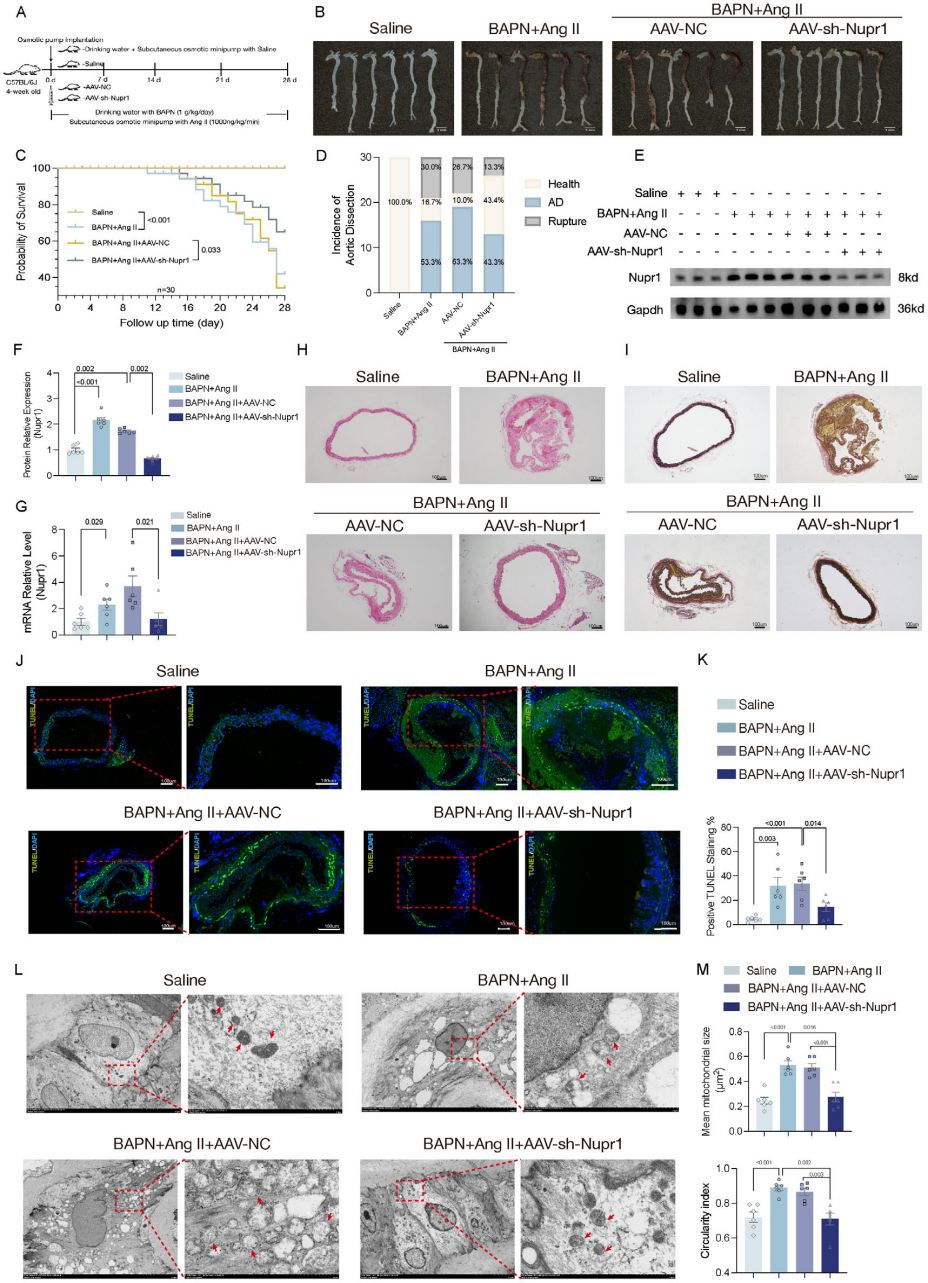
VSMC‐specific Nupr1 Deficiency Ameliorates AD Progression and Mitochondrial Apoptosis In Vivo. (A) Flow chart depicting experimental strategy. (B) Representative photographs of whole aorta from indicated groups. (C) Survival curves of the indicated groups. The rates were compared by the log‐rank test (*n* = 30 per group). (D) Incidence of AD in indicated groups (*n* = 30 per group). (E) Expression of Nupr1 in aortic tissues from indicated groups measured by Western blot. (F) Quantification of Nupr1 expression in aortic tissues from indicated groups measured by Western blot (*n* = 6 per group). (G) RT‐qPCR analysis of Nupr1 mRNA levels in aortic dissection from indicated groups. Data were presented as relative fold change to controls (*n* = 6 per group). (H) Representative macroscopic images of aorta cross‐sections stained with H&E staining. (I) Representative macroscopic images of aorta cross‐sections stained with EVG. (J) Representative images of TUNEL assay detecting apoptosis in aortic sections in indicated groups. (K) Semi‐quantitative analysis of positive TUNEL staining in aortic sections in the indicated groups (*n* = 6 per group). (L) Representative images of TEM in aortic tissues from indicated groups. (M) Quantification of circularity index and mitochondrial size in aortic tissues from indicated groups (*n* = 6 per group).

### 2.3 Ang II Drives Subcellular Redistribution of Nupr1 to Mitochondria in Apoptotic VSMCs

To gain mechanistic insight into how Nupr1 contributes to VSMC injury relevant to AD, we examined Nupr1 expression and subcellular distribution in Ang II‐stimulated VSMCs. Ang II‐stimulation significantly upregulated Nupr1 expression in VSMCs at both protein and mRNA levels (Figure 3A–C). The COMPARTMENTS resource suggested Nupr1 is localized to both the nucleus (Z‐score = 4.7) and mitochondrion (Z‐score = 4.3) with high confidence (Figure 3D). Representative immunofluorescent staining illustrated the onset of nuclear condensation (a hallmark of apoptosis) [19], and nuclear/mitochondria‐enriched Nupr1 following Ang II stimulation (Figure 3E). Quantification of compartmental Nupr1 distribution revealed slight increases in the nuclear fraction (Figure 3F) and significant increases in the mitochondrial fraction (Figure 3G), accompanied by a decrease in the cytosolic (non‐nuclear, non‐mitochondrial) fraction (Figure 3H). Furthermore, protein expression of Nupr1 increased both in the nucleus and mitochondria following Ang II stimulation (Figure 3I,J). We also treated VSMCs with the mitochondrial ROS scavenger, mito‐TEMPO, and found that scavenging primary ROS significantly attenuated the Ang II‐induced mitochondrial translocation of Nupr1 (Figure S5A). TEM images showed that Ang II stimulation induced swollen mitochondria with disrupted cristae and a reduction in nuclear area in VSMCs (Figure 3K and Figure S5B,C). However, both morphological evaluation of mitochondrial mass via Hsp60 immunofluorescence staining (Figure S5D,E) and relative mtDNA/nDNA ratio quantification (Figure S5F) confirmed that total mitochondrial mass was not significantly altered upon Ang II stimulation. In parallel, Annexin V staining confirmed increased apoptosis in VSMCs following Ang II stimulation (Figure 3L and Figure S5G). Collectively, these data indicate that Ang II promotes subcellular redistribution of Nupr1 toward mitochondria‐enriched compartments, accompanied by mitochondrial injury and apoptotic remodeling in VSMCs.

**FIGURE 3:**
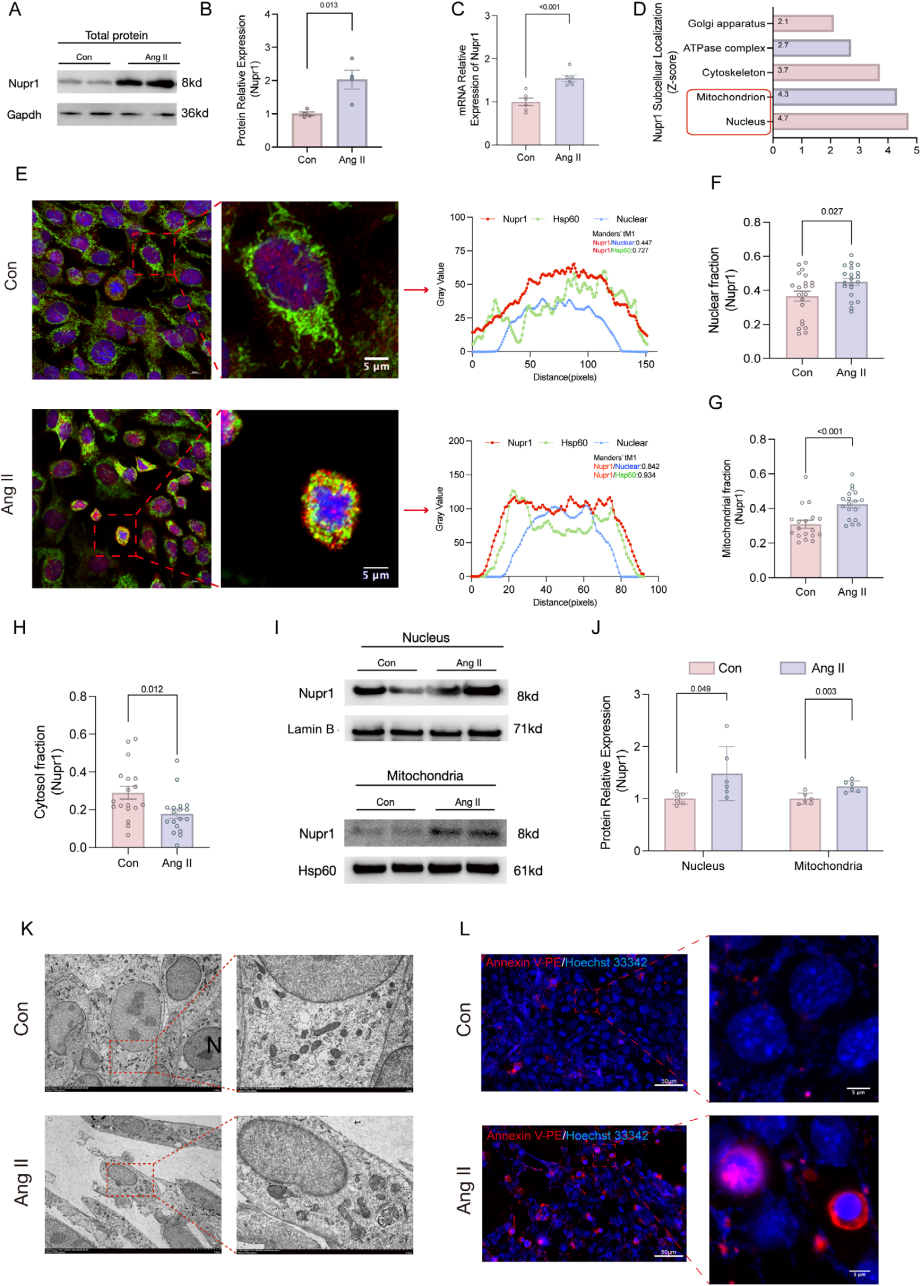
Ang II Drives Subcellular Redistribution of Nupr1 to Mitochondria in Apoptotic VSMCs. (A) Expressions of Nupr1 in total protein of VSMCs from control and Ang II‐stimulation groups measured by Western blot. (B) Quantification of Nupr1 expression in VSMCs from the indicated groups measured by Western blot (*n* = 4 per group). (C) RT‐qPCR analysis of Nupr1 mRNA levels in VSMCs from indicated groups. Data were presented as relative fold change to controls (*n* = 6 per group). (D) Subcellular localization of Nupr1 using COMPARTMENTS analysis. (E) Immunofluorescence staining revealed the cellular location of Nupr1 (red), counterstained with Hsp60 (green) and DAPI (blue). (F, G, H) Quantification of compartmental Nupr1 distribution in nuclear, mitochondria, and cytosol (non‐nuclear, non‐mitochondrial) in VSMCs (*n* = 18). (I) Expressions of Nupr1 in nuclear and mitochondria of VSMCs from the indicated groups, measured by Western blot. (J) Quantification of Nupr1 expression in VSMCs from the indicated groups measured by Western blot (*n* = 6 per group). (K) Representative images of TEM in VSMCs from indicated groups. (L) Representative images of Annexin V fluorescence, counterstained with Hoechst 33342, in indicated groups. Stronger red fluorescence represents higher apoptosis rate.

### 2.4 Genetic and Pharmacological Inhibition of Nupr1 Attenuates Ang II Induced Mitochondrial Apoptosis in VSMCs

In light of the mitochondrial enrichment of Nupr1 observed under Ang II stimulation, we hypothesized that Nupr1 acts as an important regulator of mitochondrial homeostasis and apoptotic signaling in VSMCs. Following knockdown of Nupr1 (Figure S6A–C), we observed that Nupr1 deficiency significantly protected VSMCs against Ang II‐induced cytotoxicity and reversed pathological lactate accumulation (Figure 4A,B). TEM images demonstrated that Nupr1 deficiency ameliorated the pathological mitochondrial swelling induced by Ang II stimulation, as evidenced by a reduced mitochondrial cross‐sectional area and circularity index (Figure 4C–E), alongside upregulated expression of the fusion proteins Opa1 and Mfn2 expression (Figure S6D,E). Mitochondrial bioenergetic analysis showed Nupr1 deficiency effectively mitigated impaired mitochondrial respiration in the presence of Ang II‐stimulation (Figure 4F–I). Excessive mPTP opening leads to mitochondrial dysfunction [20], which was evaluated using calcein staining in our results. Representative fluorescence images showed that Ang II reduced calcein staining and elevated Annexin‐V staining in VSMCs, which suggests excessive mPTP opening and apoptosis, whereas Nupr1 deficiency attenuated these changes (Figure 4J–L). Flow cytometric analysis showed Nupr1 deficiency under Ang II stimulation reduced early apoptosis, with no significant effect on late apoptosis (Figure 4M,N). In addition, Nupr1 deficiency reduced Bax expression and the ratio of cleaved to total caspase3, but increased the Bcl2 expression (Figure S6F,G).

**FIGURE 4:**
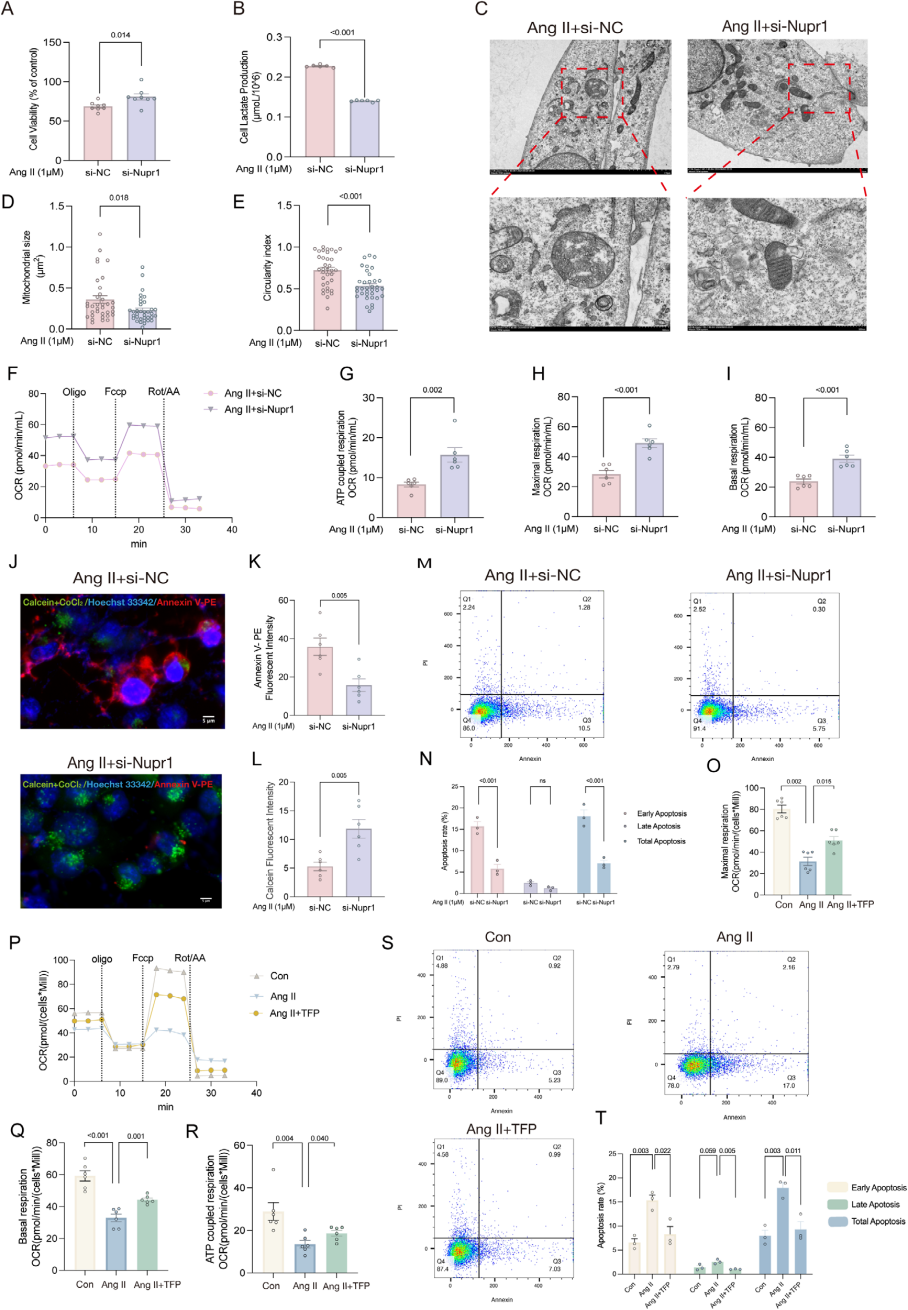
Genetic and Pharmacological Inhibition of Nupr1 Attenuates Ang II Induced Mitochondrial Apoptosis in VSMCs. (A) Cell ability was evaluated by CCK8 assay (*n* = 8 per group). (B) Lactate production of indicated groups (*n* = 6 per group). (C) Representative images of TEM in VSMCs from the indicated groups. (D) Quantification of mitochondrial size in VSMCs from indicated groups (*n* = 6 per group). (E) Quantification of circularity index in VSMC from indicated groups (*n* = 6 per group). (F) Real‐time monitoring of oxygen consumption rate (OCR) in indicated groups detected by O2k. (G–I) Quantification of ATP‐coupled respiration, mitochondrial basal respiration, and maximal respiration from indicated groups (*n* = 6 per group). (J) Representative images of calcein and Annexin V fluorescence results showing mPTP opening and apoptosis in indicated groups. Stronger green fluorescence represents a lower degree of mPTP opening, while diminished fluorescence indicates increased mPTP opening. Stronger red fluorescence represents a higher apoptosis rate. (K, L) Quantification of fluorescence intensity, including Annexin V and Calcein (*n* = 6 per group). (M) Flow cytometry analysis of Annexin and PI staining of apoptosis from indicated groups. (N) Quantification of early apoptosis, late apoptosis, and total apoptosis by flow cytometry (*n* = 3 per group). (O) Quantification of maximal respiration from indicated groups (*n* = 6 per group). (P) Real‐time monitoring of OCR in indicated groups detected by O2k. (Q, R) Quantification of ATP‐coupled respiration and mitochondrial basal respiration from indicated groups (*n* = 6 per group). (S) Flow cytometry analysis of Annexin and PI staining of apoptosis from indicated groups. (T) Quantification of early apoptosis, late apoptosis, and total apoptosis by flow cytometry (*n* = 3 per group).

To determine whether these protective effects could be pharmacologically recapitulated, we treated Ang II stimulated VSMCs with Trifluoperazine (TFP), a well‐characterized calmodulin inhibitor known to bind Nupr1 [21]. Remarkably, TFP treatment perfectly mirrored the benefits of genetic Nupr1 deficiency under Ang II stimulation. At optimal concentrations of 5 and 10 µM, TFP significantly rescued cell viability (Figure S7A). Western blot and RT‐qPCR revealed that 10 µM TFP induced a more significant downregulation of Nupr1 expression, compared to the 5 µM doses (Figure S7B–D). Consistent with the genetic model, pharmacological inhibition of Nupr1 (10 µM TFP) suppressed Ang II‐induced mPTP opening (Figure S7E,F), improved basal and maximal oxygen consumption rates, as well as ATP‐coupled respiration(Figure 4O–R), and significantly reduced apoptosis (Figure 4S,T and Figure S7G). Collectively, these findings provide robust dual evidence that targeting Nupr1 effectively relieves Ang II‐induced mitochondrial dysfunction and apoptosis in VSMCs.

### 2.5 Gadd45b is Indispensable for the Protective Effects of Nupr1 Deficiency Against Mitochondrial Apoptosis

To delineate the downstream molecular mechanisms underlying Nupr1‐mediated vascular protection, we performed transcriptomic profiling. Analysis of KEGG from transcriptomic profiling revealed that several pathways were enriched, encompassing nucleocytoplasmic transport, p53 signaling pathway, cell cycle, cellular senescence, and apoptosis (Figure 5A). Gene set enrichment analysis (GSEA) confirmed a robust suppression of apoptosis following Nupr1 deficiency (Figure 5B). Differentially expressed genes across cell‐fate pathways were mapped to a functional interaction network, which identified Gadd45b as a central node (Figure 5C). Transcriptomic quantification and subsequent RT‐qPCR validation demonstrated that Nupr1 deficiency markedly upregulated Gadd45b expression while suppressing pro‐apoptotic effectors including Caspase 9 and Bcl2l11 (Figure 5D,E). Western blot analysis corroborated the significant increase in Gadd45b expression in Nupr1‐deficient VSMCs under Ang II stimulation (Figure 5F). We next determined whether Gadd45b functionally mediated the Nupr1‐dependent protection. Strikingly, simultaneous knockdown of Gadd45b partially attenuated the anti‐apoptotic and anti‐oxidative effects of Nupr1 deficiency under Ang II stimulation, resulting in a rebound in ROS accumulation (Figure 5G,H), increased apoptotic rates, and mPTP opening (Figure 5I,J). Given this strong functional dependence, we investigated whether Nupr1 exerts its functions through direct physical interaction with Gadd45b. However, Co‐IP assays failed to detect a direct interaction between Nupr1 and Gadd45b (Figure 5K,L). Collectively, these findings demonstrate that Nupr1 deficiency protects VSMCs via a Gadd45b‐dependent mechanism. However, the lack of direct physical interaction indicates that an intermediate upstream regulator must bridge this Nupr1‐Gadd45b signaling axis.

**FIGURE 5:**
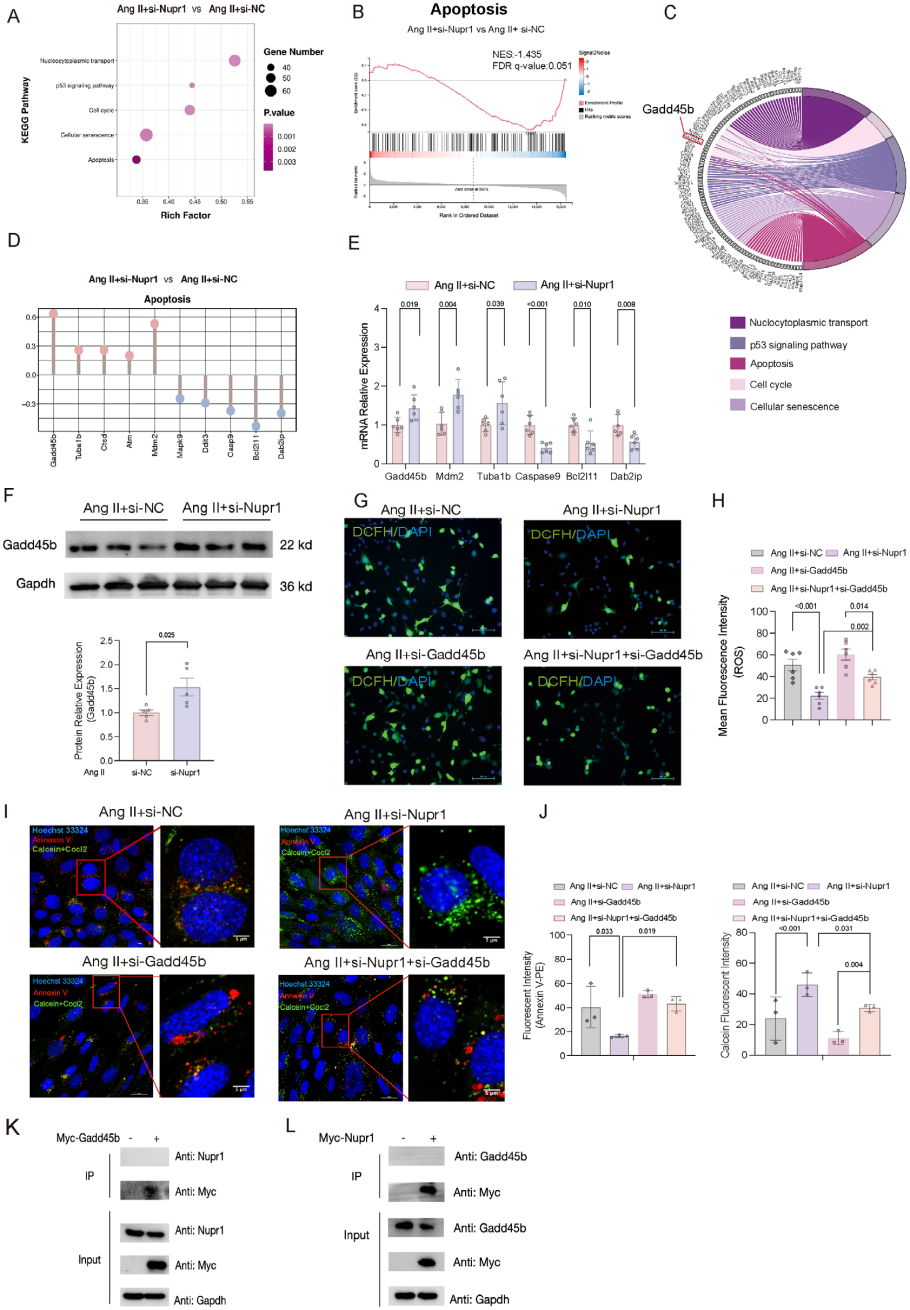
Gadd45b is Indispensable for the Protective Effects of Nupr1 Deficiency Against Mitochondrial Apoptosis. (A) Enrichment of KEGG pathways. (B) GSEA analysis in VSMCs treated with Ang II stimulation and Nupr1 deficiency. (C) Chordal diagram of GO pathways related with cell fate and the contributing DEPs. (D) Stem plot of DEPs in the apoptosis pathway from the indicated groups. (E) RT‐qPCR analysis of mRNA levels of candidate genes in VSMCs. Data were presented as relative fold change to controls (*n* = 6 per group). (F) Expressions of Gadd45b in VSMCs measured by Western blot and its quantification from indicated groups measured by Western blot (*n* = 5 per group). (G, H) Representative images of ROS staining in indicated groups. Semi‐quantitative analysis of mean fluorescence intensity in indicated groups (*n* = 6 per group). (I) Representative images of calcein and Annexin V fluorescence results showing mPTP opening in indicated groups. (J) Quantification of fluorescence intensity, including Annexin V and mPTP (*n* = 3 per group). (K) Co‐immunoprecipitation and Western blot assays of Myc‐Nupr1 and Gadd45b. (L) Co‐immunoprecipitation assays of Myc‐Gadd45b and Nupr1.

### 2.6 Nupr1 Deficiency Relieves the Spatial Sequestration of p53 to Restore *Gadd45b* Transcription

Having established *Gadd45b* as the critical but indirectly downstream effector of Nupr1, we investigated potential intermediate transcription factors bridging this regulatory axis. Given that Nupr1 lacks a classic DNA‐binding domain, we searched for intermediate transcription factors. GSEA analysis revealed a robust positive enrichment in the DNA repair (Figure 6A), a compensatory response classically orchestrated by the p53 network [22]. To test whether p53 serves as the upstream regulator, we analyzed GSEA of the p53 pathway and found a complex enrichment profile (Figure S8A). STRING database has identified p53 (Trp53) as a crucial node interacting with Gadd45b (Figure S8B). Consistent with this functional link, we identified that Gadd45b was a markedly upregulated node under Nupr1‐deficienct conditions by evaluating expression of DEGs (TPM values) in the p53 pathway (Figure 6B). Dual‐luciferase reporter assay was performed using a series of Gadd45b promoter constructs spanning different upstream regions, and we found that Nupr1 overexpression significantly suppressed Gadd45b promoter activity (Figure 6C). To gain deeper insights into the regulatory mechanisms by which Nupr1 influences Gadd45b activity, we performed JASPAR analysis and identified a highly conserved p53‐binding motif (Relative score: 0.800) within the Gadd45b regulatory region (Figure 6D). We designed specific primer sets (p1–p4), with the p4 region intentionally flanking the JASPAR‐predicted motif. ChIP‐qPCR assay demonstrated that Nupr1‐deficiency enhanced p53 occupancy at the p4 binding site of the Gadd45b regulatory region (Figure 6E). Furthermore, we validated the functional necessity of this site by replacing the predicted TFBS with a random mutant sequence (Figure 6F). Subsequent dual‐luciferase reporter assay confirmed that p53 markedly transactivated the wild‐type Gadd45b reporter, and this transactivation was significantly decreased upon transcriptional factor binding mutation (Figure 6G). Co‐IP assay confirmed an interaction between Nupr1 and p53 (Figure 6H). AlphaFold3 structural modeling predicted specific binding pockets and several hydrogen bonds between Nupr1 and p53 (including Nupr1‐Y30 and p53‐H230, Nupr1‐E26 and p53‐Q112, Nupr1‐D25 and p53‐G114) (Figure 6I). To validate these structural predictions, point mutation plasmids were constructed in which the predicted key residues (D25, E26 and Y30) of Nupr1 were individually mutated to Alanine (D25A, E26A and Y30A). Compared with wild‐type Myc‐Nupr1, the D25A and E26A mutants maintained a detectable interaction with p53, whereas the Y30A mutation virtually eliminated this co‐precipitation (Figure 6J).

**FIGURE 6:**
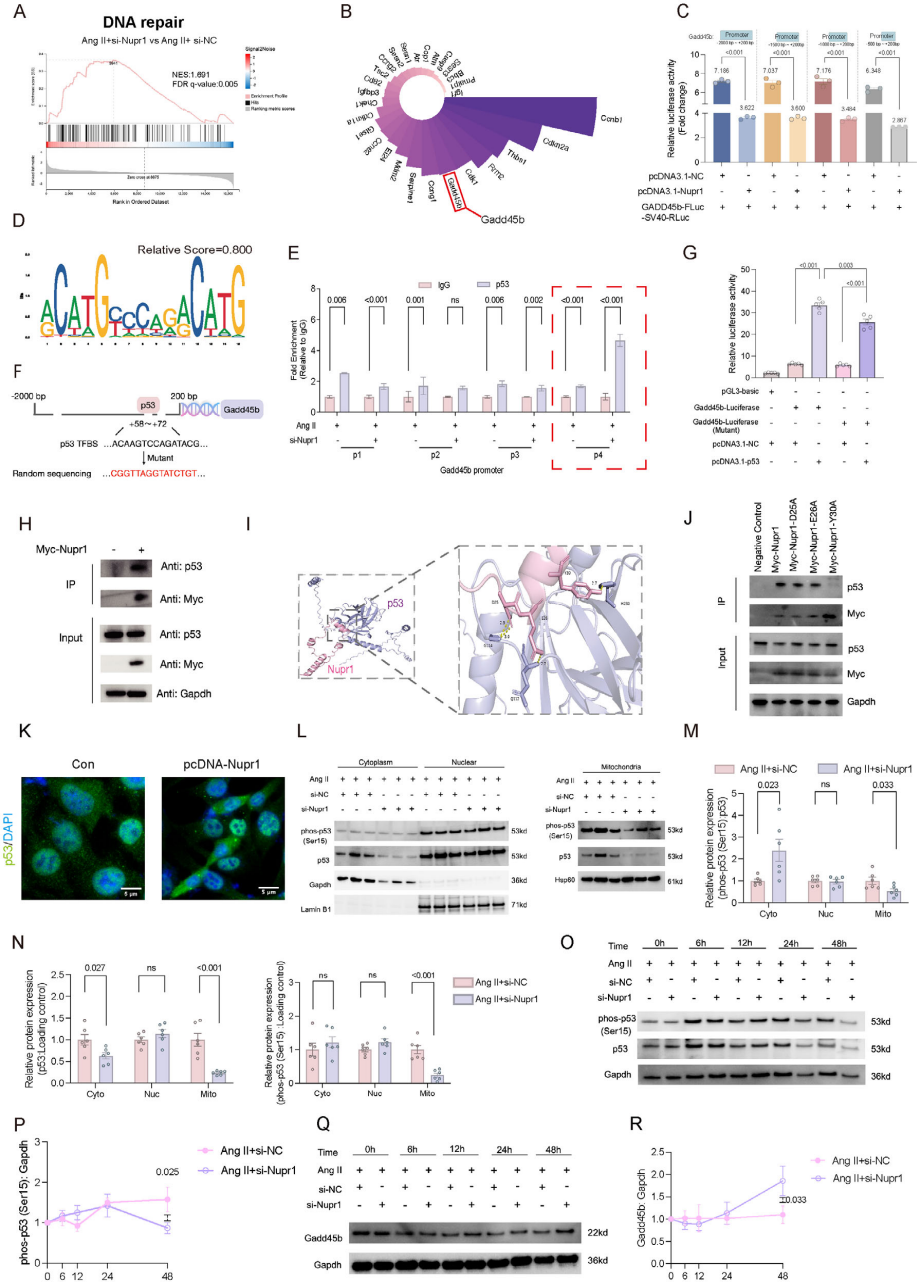
Nupr1 Deficiency Relieves the Spatial Sequestration of p53 to Restore *Gadd45b* Transcription. (A) GSEA analysis of DNA repair. (B) Rose plot of p53 pathway and enriched genes. (C) A dual‐luciferase reporter assay was conducted to compare the Nupr1 binding element and the activities of the Gadd45b promoter with different regions (*n* = 3 per group). (D) Predicted p53 binding Gadd45b promoter motifs by Jaspar. (E) The binding level of p53 at the predicted site was validated by ChIP‐qPCR. (F) Flow chart depicting mutations of p53 binding site. (G) A dual‐luciferase reporter assay was conducted to compare the p53 binding element and the activities of the Gadd45b regulatory region with mutations (*n* = 5 per group). (H) Coimmunoprecipitation and Western blot assays of Myc‐Nupr1 and p53. (I) The binding sites of Nupr1 and p53 proposed by AlphaFold3. Nupr1 molecule was shown as light pink sticks, and p53 was shown as light purple sticks. (J) Coimmunoprecipitation and Western blot assays of Myc‐Nupr1‐mutations and p53. (K) Representative images of the subcellular location of p53 in VSMCs from indicated groups. (L) Expression of phos‐p53 (Ser15) and p53 in the cytoplasm, nuclear and mitochondria from the indicated groups, measured by Western blot. (M, N) Quantification of phos‐p53 (Ser15) and p53 expression, and ratio of phos‐p53/p53 in VSMCs from indicated groups measured by Western blot (*n* = 6 per group). (O) Expressions of phos‐p53and p53 over 48 h in VSMCs from the indicated groups, which were measured by Western blot (*n* = 6 per group). (P) Quantification of phos‐p53 expression in VSMCs from indicated groups and analyzed by Two‐Way ANOVA. (Q, R) Expression and quantification of Gadd45b over 48 h in VSMCs from indicated groups measured by Western blot (*n* = 6 per group) and analyzed by Two‐Way ANOVA.

Based on these observations, we hypothesized that Nupr1 negatively regulated transcriptional activation of Gadd45b by mediating p53 function. Given that p53 function relies heavily on its subcellular localization [23], we investigated the effects of Nupr1 overexpression on p53 spatial retention. While p53 exhibited a diffuse distribution under unstressed conditions, overexpression of Nupr1 robustly induced its cytoplasmic retention (Figure 6K and Figure S8C). We next performed subcellular fractionation to determine whether this cytoplasmically retained p53 translocated to the mitochondria under pathological stress to trigger transcription‐independent apoptosis. This analysis revealed that, under Ang II stimulation, Nupr1 deficiency significantly attenuated the accumulation of p53 and its phosphorylated forms (Ser15, Ser20, and Ser46) in the mitochondria, along with a decrease in p53 and p‐p53 (Ser46) in the cytoplasm. However, within the nucleus, Nupr1 deficiency selectively suppressed the pro‐apoptotic marker (Ser46) [24], while the levels of p53 and the stabilization markers p‐p53 (Ser15, Ser20) [25] showed no significant alterations (Figure 6L–N and Figure S8D–I).

Beyond spatial sequestration, the functional output of p53 is also intricately governed by its temporal activation profile. Accordingly, temporal dynamic analysis demonstrated that Ang II induced a biphasic response in phosphorylation modifications of p53. Specifically, the levels of phos‐p53(Ser15) initially peaked at 6 h, exhibited a transient decline at 12 h, and were followed by a sustained elevation up to 48 h. Although Nupr1 deficiency did not alter the overall bi‐phasic pattern of the phos‐p53: p53 ratio, it effectively facilitated the attenuation of the absolute p53 and phos‐p53(Ser15) burden at the later stage (48 h) (Figure 6O,P and Figure S8J,K). Notably, Nupr1 deficiency induced subtle fluctuations during the initial 24 h, but it enabled a progressive accumulation of Gadd45b until 48 h (Figure 6Q,R). Collectively, these findings demonstrated that Nupr1 acts as a pathological switch, structurally sequestering p53 subcellular location and altering temporal dynamics to prevent the protective role of Gadd45b.

### 2.7 Gadd45b Overexpression Rescues Mitochondrial Apoptosis and Blunts AD

To definitively establish the therapeutic potential of this downstream effector of Nupr1, we evaluated the efficacy of Gadd45b overexpression across both in vitro and in vivo models. In Ang II‐stimulated VSMCs, Gadd45b overexpression significantly improved mitochondrial bioenergetics, evidenced by increased basal, ATP‐coupled, and maximal respiration oxygen consumption rates (Figure S9A–D). Furthermore, compared to Ang II treatment, Gadd45b overexpression mitigated excessive mPTP opening and reduced mitochondrial calcium influx, restoring mitochondrial membrane potential as indicated by calcein, Rhod‐2AM and Mito‐Tracker staining, respectively (Figure S9E–H). Consequently, Annexin‐V (Figure S9E,F) and TUNEL (Figure S9I) staining revealed that Gadd45b overexpression mitigated Ang II‐induced apoptosis.

Above the cellular protections of Gadd45b overexpression under Ang II stimulation in VSMCs, we next investigated the translational value of Gadd45b in the BAPN+Ang II‐induced murine AD model. AAV‐Gadd45b was applied in mice (*n* = 30 each group) on the first day, and they were treated with BAPN and Ang II for 28 days (Figure 7A). Strikingly, Gadd45b overexpression conferred a profound survival advantage, reducing the mortality rate from 40% (12 of 30) to 16.7% (5 of 30) and the overall AD incidence from 86.7% (26 of 30) to 50.0% (15 of 30), compared to BAPN+Ang II+AAV‐NC controls (Figure 7B,C). Macroscopic examination further confirmed that AAV‐Gdd45b administration significantly relieved the severity of AD compared to that in controls (Figure 7D). Blood pressure monitoring showed no significant difference in systolic blood pressure between the BAPN+Ang II+AAV‐NC and BAPN+Ang II+AAV‐Gadd45b groups (Figure 7E), suggesting that the protective effects of Gadd45b is largely independent of systemic blood pressure. The transduction efficiency of AAV‐Gadd45b was validated by Western blot and RT‐qPCR (Figure 7F,G). HE and EVG staining revealed that AAV‐Gadd45b remarkably mitigated vascular wall destruction and disruption in elastin alignment of AD model (Figure 7H–J). Mirroring the functional mitochondrial protection observed in vitro, TEM images showed AAV‐Gadd45b treatment alleviated pathological mitochondrial alterations in aorta sections, effectively reversing organelle swelling and impairment of mitochondrial double membrane integrity (Figure 7K–M). Furthermore, this targeted treatment prevented apoptosis‐related nuclear condensation (Figure 7N). Finally, in vivo TUNEL staining revealed that AAV‐Gadd45b alleviated BAPN+Ang II‐induced VSMCs apoptosis in the media (Figure 7O,P). Together, these multi‐level evaluations indicate that Gadd45b overexpression is sufficient to rescue mitochondrial function and blunt AD pathogenesis.

**FIGURE 7:**
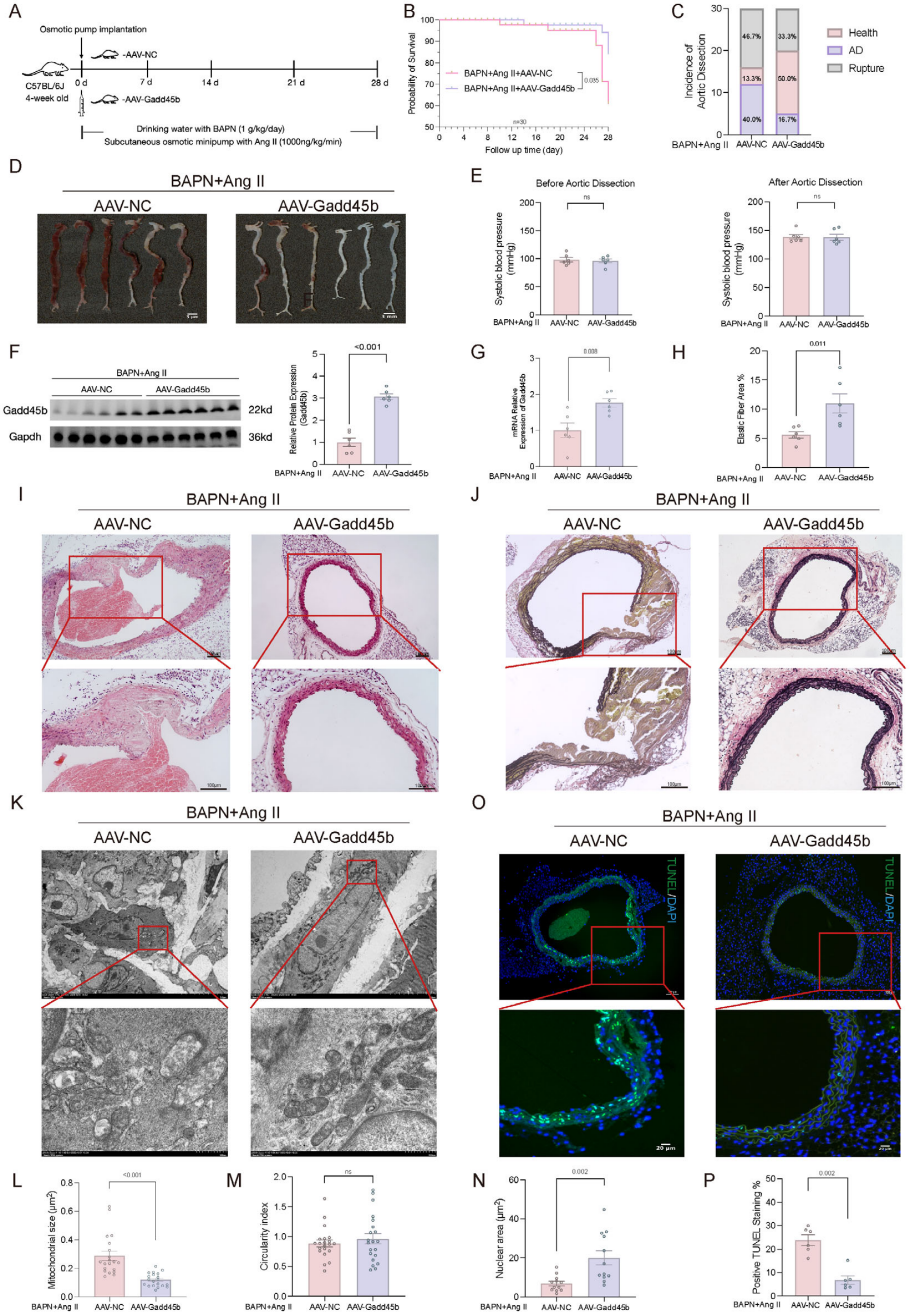
Gadd45b Overexpression Rescues Mitochondrial Apoptosis and Blunts AD. (A) Flow chart depicting experimental strategy for NC or Gadd45b using AAV delivery. (B) Survival curves of the indicated groups. The rates were compared by the log‐rank test (*n* = 30 per group). (C) Incidence of AD in indicated groups (*n* = 30 per group). (D) Representative photographs of the whole aorta from indicated groups. (E) Blood pressure measurements (*n* = 6 per group). (F) Expression and quantification of Gadd45b in aortic tissues from indicated groups measured by Western blot (*n* = 6 per group). (G) RT‐qPCR analysis of Gadd45b mRNA levels in aortic dissection from indicated groups. Data were presented as relative fold change to controls (*n* = 6 per group). (H) Quantitative analysis of elastic fibers in indicated groups (*n* = 6 per group). (I) Representative macroscopic images of aorta cross‐sections stained with H&E staining. (J) Representative macroscopic images of aorta cross‐sections stained with EVG. (K) Representative images of TEM in aortic tissues from indicated groups. (L–N) Quantification of circularity index (*n*=21 per group), nuclear area (*n*=12 per group), and mitochondrial size (*n*=20 per group) in aortic tissues from indicated groups. (O) Representative images of TUNEL assay detected apoptosis in aortic sections in the indicated groups. (P) Quantitative analysis of positive TUNEL staining in aortic sections in indicated groups (*n* = 6 per group).

## 3 Discussion

Our study delves into the complexity of AD, with a particular emphasis on the crucial role of apoptosis. Initially guided by morphological observations and single‐cell RNA‐seq analysis, we identified Nupr1 as a key driver of this apoptotic cascade. A key finding is the pathological accumulation of Nupr1 at the mitochondria triggered by Ang II‐induced mitochondrial ROS. This subcellular shift underscores a pivotal role for Nupr1 in triggering mitochondrial dysfunction, highlighting its essential contribution to AD progression. Nupr1 has been previously implicated in driving pathological vascular remodeling through its conventional transcriptional regulation [12]. In contrast to this canonical role, Nupr1 functions here as a pathological spatial switch. Serving as a physical anchor, it tethers p53 to the mitochondrial network. This spatial sequestration simultaneously triggers transcription‐independent mitochondrial apoptosis and suppresses the protective Gadd45b function. These unique spatiotemporal characteristics highlight a novel subcellular mechanism driving AD progression.

We identified that *Nupr1* was significantly enriched within cell‐fate pathways and confirmed its increased expression in both human and murine AD tissues compared to non‐AD controls. VSMC‐specific deficiency of Nupr1 preserved elastin integrity, attenuated mitochondrial dysfunction, relieved apoptosis, and alleviated AD formation and rupture in vivo. To decipher the downstream molecular events driving this apoptotic phenotype, we utilized Ang II stimulation to effectively recapitulate pathological vascular stress in vitro, which was consistent with classical studies [26, 27]. In this context, Nupr1 may act as a critical amplifier, driving a subsequent ROS burst via the ROS‐induced ROS release (RIRR) cascade [28, 29]. This oxidative damage aligns with our transcriptomic profiling, which showed that Nupr1 deficiency is positively correlated with DNA repair processes and negatively correlated with apoptosis in VSMCs, the two key features of AD [30, 31]. Collectively, these findings indicate that Nupr1 is a pivotal regulator of AD development.

A key determinant of cell fate during cellular stress responses is commonly referred to as the dynamics of p53. Although p53 dynamics were initially characterized as variations of total protein levels, it's now established that its activity and spatial location dictate whether a stressed cell initiates adaptive repair or apoptosis [32]. Crucially, our results have identified that Nupr1 is sufficient to drive the cytoplasmic sequestration of p53, establishing Nupr1 as a cytoplasmic anchor for p53. Furthermore, we observed that Nupr1 deficiency during Ang IIstimulation reduced the non‐nuclear retention of p53, especially in mitochondria. In line with previous conceptual models [33, 34], these findings support our results that relieving the Nupr1‐mediated spatial blockade effectively prevented p53 mitochondrial accumulation. Interestingly, this decrease of p53 from the mitochondria did not trigger a compensatory nuclear p53 surge. This functionally mirrors Pifithrin‐µ, a classic inhibitor that averts apoptosis by blocking p53 mitochondrial binding while preserving its nuclear transcriptional capacity [35, 36]. Consequently, we postulated that blocking p53 mitochondrial accumulation may mitigate the pathological stress response, thereby restoring the Gadd45b‐mediated survival response without requiring hyperactive nuclear p53 activation.

Building on this speculation, we provided molecular evidence for the direct crosstalk between p53 and Gadd45b by demonstrating that p53 directly binds to the Gadd45b regulatory region and subsequently leads to the transcriptional activation of Gadd45b. Gadd45b is classically recognized as a stress‐inducible sensor pivotal for DNA repair and cell cycle arrest [37]. Numerous studies have focused on its protective functions in nervous system resilience and myocardial ischemic injury [38, 39]. This aligns with our findings regarding its anti‐apoptotic role in VSMCs, where overexpression of Gadd45b effectively attenuated mitochondrial dysfunction‐induced apoptosis in vitro. Additionally, AAV‐mediated overexpression of Gadd45b in VSMCs sufficiently rescued mitochondrial integrity, suppressed apoptosis, and blunted AD progression in vivo. Although some reports suggest that pathological upregulation of Gadd45b may aggravate hypoxia‐induced apoptosis in cardiomyocytes [39, 40], this functional divergence underscores the highly context‐dependent and tissue‐specific nature of Gadd45b responses. Therefore, rather than a generic stress response, our findings establish Gadd45b as the indispensable functional effector of this p53‐dependent survival axis; and the restoration of Gadd45b is a fundamental requirement for preserving VSMC mitochondrial function against stressed injury.

Having established the indispensable role of Nupr1 in the survival axis, our study highlights Nupr1 as a disease‐specific upstream target for AD prevention. Modulating this upstream switch provides a significantly safer therapeutic strategy, given that directly targeting downstream effectors like p53 is precluded by catastrophic oncogenic risks and trial failures [41, 42]. Herein, we confirmed the translational potential by utilizing an SM22α‐targeted AAV to achieve precise VSMC‐specific Nupr1 deficiency, which effectively blunted AD progression in vivo. However, translating this genetic success into a pharmacological approach remains challenging. For instance, the systemic application of TFP is intrinsically precluded by its profound central nervous system side effects [43, 44]. Furthermore, current Nupr1‐targeted compounds are mechanistically insufficient. ZZW‐115, a compound designed specifically to restrict Nupr1 nuclear translocation [45], failed to mitigate pathological vascular remodeling in vivo [46]. This failure inadvertently validates our spatial sequestration mechanism that Nupr1 deficiency mitigates AD by preventing the aberrant mitochondrial accumulation of p53, thereby restoring the protective Gadd45b survival response. Consequently, agents merely restricting nuclear translocation fail to abrogate this fatal mitochondrial signaling. Therefore, future precision therapeutics should focus on disrupting the interaction between Nupr1 and p53 at the mitochondrial network, offering a targeted approach for AD prevention.

## 4 Conclusions

In conclusion, our findings redefine Nupr1 not merely as a canonical transcription cofactor, but as a critical pathological spatial switch in AD pathogenesis. We revealed that the Nupr1‐mediated mitochondrial sequestration of p53 attenuates the Gadd45b‐dependent survival response, precipitating a fatal mitochondrial apoptosis cascade. Moving forward, targeting this specific spatial sequestration to reactivate the endogenous survival program presents a novel therapeutic approach for the prevention and treatment of AD.

## 5 Methods

### 5.1 Human Subjects and Specimens

The experiments conducted with human aortic tissue samples were performed in compliance with ethical principles of the Declaration of Helsinki. The protocol was approved by the local institutional ethics committee (2024KY081). Written informed consent was obtained from all patients or the donors’ families before specimen collection. The diseased aortic tissue samples were obtained from the aortic rupture regions in AD patients who underwent aortic surgery (*n* = 18), and corresponding control aortic tissue samples were obtained from donors of heart transplant donors (*n* = 18). The patients were excluded if they had a heritable form of Marfan syndrome. The aorta status of all subjects was evaluated using at least one imaging examination, i.e., echocardiography or computed tomography angiography (CTA). The detailed information of AD patients and donors was presented in Table S1. Samples for immunofluorescent staining were processed for paraffin embedding and cut to 4‐µm thickness. Samples for western blot were stored at −80°C.

### 5.2 Animal Studies

C57BL/6J mice were provided from Shanghai Slac Laboratory Animal Co., Ltd (SHDSYY‐2020‐1734). All animals used for the study were male for less sex hormonal variation and high AD incidence. C57BL/6J mice, AAV‐sh‐Nupr1 and AAV‐NC, AAV‐Gadd45b mice were fed with β‐aminopropionitrile (BAPN, 1 g/kg/day, A0408, TCI, Japan) in drinking water and infused with Ang II (GP10023, GLP Bio, 1000 ng/kg/min) by mini‐osmotic pumps (Model 2004, Alzet) for 28 days to induce AD. Mice were kept in a specific pathogen‐free animal room (21°C ± 2°C, 45%–65% humidity) under a 12 h light/dark cycle, with free access to food and water. Four weeks later, the mice were anesthetized intraperitoneally with 1% pentobarbital sodium for sample collection. All animal experiments were conducted in compliance with the National Institutes of Health Guidelines on the Care and Use of Laboratory Animals (National Institutes of Health Publication, eighth edition, 2011) and approved by the Animal Ethics Committee of the local institutional (Code: 2022‐Y‐0763).

### 5.3 Construction and Delivery of Adeno‐Associated Virus (AAV) Vectors

To knock down Nupr1 in aortic VSMCs, HBAAV2/9‐Sm22α‐mir30‐m‐Nupr1‐mCherry was constructed and packaged by Han Biosciences (Shanghai, China). Additionally, Gadd45b was subcloned into an AAV9 vector harboring the Sm22a promoter to generate the recombinant AAV9‐Sm22a‐Gadd45b plasmid. This plasmid, together with the AAV2/9n (MiaoLing Plasmid Platform, China) helper plasmid and pAd‐ΔF6 (MiaoLing Plasmid Platform, China) packaging vector, was co‐transfected into AAV293 cells using lipo‐293 plus transfection reagent (Beyotime, Cat. No. C0522). Recombinant AAV9 was prepared following previously established protocols [47]. Viral titer of the AAV9 was quantified by quantitative polymerase chain reaction (qPCR) using primers targeting the woodchuck hepatitis virus posttranscriptional regulatory element (WPRE). Four‐week‐old C57BL/6J mice were randomly grouped before administering a single dose of 1.8×10^12^ vector genomes of AAV‐sh‐Nupr1 or AAV‐NC, and 5 × 10^11^ vector genomes of AAV‐Gadd45b and AAV‐NC via tail vein injection per mouse at first day.

### 5.4 Cell Culture and Transfection

The mouse aortic smooth muscle cells (MOVAS, BNCC Biotech) were maintained in DMEM supplemented with 10% fetal bovine serum and 1% penicillin (100 U/mL)/streptomycin (100  µg/mL). The cells were treated with 1 µM Ang II for 48 h. Small interfering RNA (siRNA) targeting Nupr1 (si‐Nupr1) and the corresponding NC (si‐NC) were designed and synthesized by HaiXing Biotechnology (Fujian, China). siRNA targeting Gadd45b (si‐Gadd45b) and the corresponding NC (si‐NC) were designed and synthesized by Han Biosciences (Shanghai, China). Transfection of siRNA was performed with Lipofectamine 8000 (Beyotime) for 48 h in accordance with the manufacturer's manual. The sequences of Nupr1‐specific siRNA‐Nupr1 and siRNA‐Gadd45b were listed in Table S2. Trifluoperazine (TFP) was purchased from MedChemExpress (HY‐B0532A, China) and added to the culture medium at the 10 µM concentrations for 24 h before cells were harvested for further analysis. Mito‐Tempo was purchased from MedChemExpress (HY‐112879, China) and added to the culture medium at the 1 µM concentrations for 24 h.

### 5.5 Protein Extraction and Western Blot

Whole‐cell lysates were acquired by resuspending cell pellets in ice‐cold RIPA buffer with freshly prepared proteinase inhibitor, phosphatase inhibitors, and PMSF. The nuclear protein and cytoplasmic protein extractions were conducted following the manufacturer's instructions for the Nuclear and Cytoplasmic Protein Extraction kit (P0027, Beyotime, China). The mitochondrial protein extractions were conducted following the manufacturer's instructions for the Mitochondrial Isolation and Protein Extraction Kit (PK10016, Proteintech). Equal amounts of protein (20–30 µg) from samples were loaded and separated on SDS‐polyacrylamide gels and were transferred onto poly‐vinylidene fluoride (PVDF) membrane by electrophoretic transfer. The membranes were blocked with 5% BSA for 2 h and incubated with specific primary antibodies at 4°C overnight. Then the membranes were incubated with corresponding HRP‐conjugated secondary antibodies for 2 h at room temperature. The protein bands were visualized by enhanced chemiluminescence (Millipore) using the ChemiDoc MP system (Bio‐Rad), and band densities were quantified by densitometry using an Image J software. The details of antibodies were listed in Table S3.

### 5.6 Co‐Immunoprecipitation

VSMCs were washed with ice‐cold PBS, lysed with 500 µL lysis buffer with protease inhibitor cocktail. After centrifugating to obtain the lysates at 4°C, 50 uL supernatant was aliquoted for input, and the remaining supernatant was preincubated with 20 µL  Anti‐Myc‐beads (P2183S, Beyotime) overnight at 4°C. VSMCs transfected with empty vector were used as controls. The immunocomplexes were washed 4 times with lysis buffer to discard nonspecifically bound protein, and precipitated protein was eluted from beads by resuspending in 1X SDS‐PAGE loading buffer and boiling for 10 min. Both input and immuno‐precipitates were analyzed by immunoblotting.

### 5.7 Immunofluorescence Staining

Aortas isolated from mice were fixed with 4% paraformaldehyde. After dehydration with 20% sucrose, aortas were embedded in wax and sliced with 4 µm tissue sections. Sections were stained with were incubated with antibodies to specifically detect Nupr1 (1:100, Immunoway, YT8077), α‐SMA (1:100, Boster, BM0002), and CD31 (1:100, Abcam, ab9498), CD68 (1:100, Abcam, ab955), F4/80 (1:100, Abcam, Ab300421), PDGFα (1:100, Proteintech, 84383‐2‐RR). VSMCs were fixed with 4% paraformaldehyde and treated with 0.3% TritonX‐100, then incubated with antibodies to specifically detect Hsp60 (1:100, AF0186, Beyotime), p53 (1:100, 10442‐1‐AP, Proteintech). Followed by the secondary Alexa Fluor 488‐conjugated Goat anti‐mouse (1:200, A‐11029, Invitrogen), Goat anti‐Rabbit IgG (H+L) Cross‐Adsorbed Secondary Antibody, TRITC (1:200, T‐2769, Invitrogen). Nuclei were counterstained with DAPI (C1006, Beyotime) or Hoechst 33342 (C1027, Beyotime). Sections were imaged using a Nikon microscope (MODEL ECLIPSE NI‐U, Japan) and Nikon confocal microscopy (Nikon AXR, NSPARC, Japan).

### 5.8 Flow Cytometric

Cells after different treatments were digested down with EDTA‐ free trypsin, centrifuged, and 1–5 × 10^5^ cells were collected, washed with cooled PBS, and resuspended in Binding Buffer. Annexin V‐ FITC and PI dye were added separately and incubated for 20 min at room temperature (40302ES50, Yeasen). Finally, detection was performed by flow cytometry (FACSCanto ^(TM)^ II, BD).

### 5.9 Measurement of Lactate Levels

Lactate levels were detected in the supernatant of VSMCs after different treatments. Cells were lysed in extraction buffer at 4°C and then centrifuged at 12 000xg for 10 min, followed by collection of the supernatant. The absorbance values of the supernatant and standard solutions were measured at 570 nm via a colorimetric lactate content assay kit (BC2235, Solarbio Life Science) according to the manufacturer's protocol. The lactate concentration was calculated via a standard curve.

### 5.10 Hematoxylin and Eosin Staining and Elastic Fiber Staining

Aortas isolated from mice were fixed with 4% paraformaldehyde. After dehydration through graded ethanol, aortas were embedded in wax and sliced with 4 µm tissue sections. Sections were stained with Elastin Van Gieson (EVG) and hematoxylin‐eosin (HE). Sections were imaged using a Nikon microscope (MODEL ECLIPSE NI‐U, Japan). Representative histological photomicrographs are presented. Quantification of elastic fiber was calculated by Image J.1.51.

### 5.11 Real‐Time Quantitative RT‐polymerase Chain Reaction (PCR) and RNA‐Sequencing

Total RNA was isolated using TRIzol and then used for cDNA synthesis using a PrimeScript RT reagent kit (Takara RR370A, Japan). mRNA expression was measured by Real‐time quantitative PCR with SYBR Premix Ex Taq (Takara, RR820A, Japan) in a LightCycler 480 Real‐time PCR System (Roche Applied Science). GAPDH was used as the housekeeping genes. Total genomic DNA was extracted from VSMCs according to the manufacturer's protocol (Yeasen, 18700ES50, Shanghai, China). Primer information is presented in Table S4.

For RNA‐sequencing, cell lysis was extracted from VSMCs treated with Ang II and co‐treated of Ang‐ and si‐Nupr1 in the condition of ice bath and washed with DEPC‐treated water. Then, the lysis was collected for isolation of total RNA and generation of a cDNA library. RNA‐sequencing was conducted by a sequencing service contractor (BGI Technology Co., Ltd., Shenzhen, China). Differentially expressed genes (DEGs) were selected using the R Bioconductor software package edge R. False discovery rate < 0.05 and fold change > 2 or < 0.5 were set as the cut‐off criteria used to identify DEGs.

### 5.12 TUNEL Assay Staining

To measure apoptosis in aortas, a cell death detection kit (#C1088; Beyotime) was used to perform TUNEL staining in accordance with the manufacturer's instructions. First, aortic sections were fixed with 4% paraformaldehyde, permeabilized with 0.3% Triton X‐100, and subjected to TUNEL assay staining. Apoptotic cells were detected as green fluorescence, and the nuclei were labeled with DAPI. Tissue sections were observed under a Nikon microscope (MODEL ECLIPSE NI‐U, Japan). Fluorescence intensity was quantified using ImageJ software.

### 5.13 Dual‐Luciferase Reporter Assay

Two dual‐luciferase experiments were used in this study. One was conducted to verify the relationship between Nupr1 and Gadd45b. Another dual‐luciferase activity assay was conducted to verify the relationship between p53 and Gadd45b. Plasmids encoding different region promoter of Gadd45b, Nupr1 plasmids, and p53 plasmids were transfected into 293T cells. The luciferase activity was then measured using a dual luciferase assay kit (11402ES60, Yeasen) according to the manufacturer's instructions. The R‐Luciferase vector (pRL‐TK) was used as an internal reference.

### 5.14 Mitochondrial Respiration Analysis

Mitochondrial respiration in VSMCs was analyzed using a mitochondrial function detection system (Oroboros, Oxygraph‐2k, Austria). Briefly, VSMCs were cultured with medium added 1 µmol/L Ang II and treated with or without plasmids for 48 h. The oxygen consumption rate (OCR, pmol/min) was monitored in real time, with sequential treatments with oligomycin (ATP synthase inhibitor), FCCP (mitochondrial uncoupler), and rotenone/antimycin A (respiration inhibitor) to evaluate OCR from proton leak, maximum respiration capacity, and nonmitochondrial respiration, respectively. OCR was recorded for 3 consecutive measurement cycles at each stage, and the average values were calculated.

### 5.15 Mitochondrial Permeability Transition Pore Assay

According to mPTP test kit instructions (C2009S, Beyotime), 200 µL of Calcein AM staining solution and fluorescence quenching working solution were added to each well after the respective cell treatments. After incubating for 40 min at 37°C, the staining solution was removed, and the preheated medium was added for an additional 30 min incubation before washing with PBS. Subsequently, 4% paraformaldehyde was added for fixation, incubating for 40 min, followed by PBS washing. Finally, samples were mounted with an anti‐fluorescence quencher (36307ES08, Yeasen) and observed using a Nikon microscope (MODEL ECLIPSE NI‐U, Japan).

### 5.16 Reactive Oxygen Species (ROS) Measurement

Intracellular ROS level was measured by the ROS Assay Kit (S0033, Beyotime). After treatment with si‐Nupr1 and si‐Gadd45b for 48 h, the cells were incubated with 10 µM DCFH‐‐DA in serum‐free medium at 37°C for 20 min in the dark. After the cells were washed 3 times with PBS, images were observed under a Nikon microscope (MODEL ECLIPSE NI‐U, Japan).

### 5.17 PPI Analysis

To investigate the interactions of Gadd45b and its related proteins, we constructed the Gadd45b‐related PPI networks based on the STRING database (https://www.string‐db.org) with a high confidence score of 0.7. We used Cytoscape software for PPI network visualization and applied the cytoHubba plugin in Cytoscape to calculate the network modules.

### 5.18 ELISA

Serum Nupr1 levels were determined using a double‐antibody sandwich Elisa according to the manufacturer's instructions (Nupr1, Coibo Bio, Shanghai, CB15645‐Mu). Briefly, 50 µL serum of the test samples was added and incubated for 1 h at 37°C. Subsequently, the biotin‐antibody and HRP‐avidin were added and incubated for 1 h at 37°C, respectively. Finally, the substrate solution and termination solution were added in succession and read at an OD of 450 nm by MOLECULAR DEVICES (SpectraMax Id3).

### 5.19 CCK8

Cell viability was determined using Cell Counting Kit‐8 (K1018, APE) according to the manufacturer's instructions. Briefly, cells were treated with si‐Nupr1 for 48 h or with TFP for 24 h, and then 10 µL CCK‐8 was added to each well. After 2 h incubation at 37°C, the absorbance was read at 450 nm using a microplate reader (MOLECULAR DEVICES, SpectraMax iD3).

### 5.20 Transmission Electron Microscopy (TEM) Analysis

Aortic tissues and cell pellets were added to 2.5% glutaraldehyde and fixed at 4°C for 1day. Cell samples were washed three times with PBS, followed by gradient dehydration, embedding, and finally ultrathin sectioning (50–100 nm) using an ultrathin sectioning machine (EM UC7, Germany). Specimens were double‐stained with 3% uranyl acetate and lead citrate, observed, and photographed under a transmission electron microscope (Hitachi, Japan).

### 5.21 Jaspar

The potential p53‐binding motifs within the Gadd45b promoter region were predicted by JASPAR, and schematic images of the potential p53‐ binding sites in the Gadd45b promoter region are shown.

### 5.22 Visualization of AlphaFold3 Prediction

AlphaFold3‐predicted Nupr1‐p53 structures were visualized using PyMOL software. Protein interfaces were colored light pink for Nupr1‐peptide interfaces and light purple for p53‐peptide interfaces.

### 5.23 ChIP‐qPCR

ChIP experiments were performed following the manufacture of ChIP assay kit (Beyotime, P2080S) described. In brief, 1 × 10^7 ^cells were fixed in 1% formaldehyde for 10 min at room temperature and sonicated to shear the chromatin. Immunoprecipitation of crosslinked chromatin was performed overnight at 4 °C with antibodies. An equal amount of isotype immunoglobulin G (IgG) was used as a background control. Primers for ChIP‐qPCR are shown in Table S4.

### 5.24 Statistical Analysis

All quantitative data were presented as mean ± SEM from at least 3 independent experiments using individual animals or independent cell cultures (biological replicates). Statistical analysis was performed using GraphPad prism 10.0 software (GraphPad Software, San Diego, CA, USA). The normality of the dataset generated in this study was assessed using the Shapiro–Wilk test. For two‐group comparisons with normally distributed data, a two‐tailed unpaired Student's *t*‐test was performed. For data that did not conform to a normal distribution, nonparametric statistical analysis (Mann–Whitney U test) was used. For the analysis of protein expression across different treatments and time points, a two‐way analysis of variance (ANOVA) followed by Sidak's post hoc test was used for multiple comparisons. Animal survival rates were analyzed using the Kaplan–Meier method, and differences between survival curves were evaluated using the Log‐rank (Mantel‐Cox) test. Values were considered significant at *p* < 0.05.

## Author Contributions

**Xi Yang**: conceptualization, writing – original draft, writing – review and editing, methodology, formal analysis, investigation, validation, data curation. **Ke‐Yuan Chen**: software, methodology, investigation, validation, writing – review and editing, data curation, conceptualization. **Yue Tian**: software, data curation, investigation, validation. **Zong‐Sheng Jiang**: validation, methodology, formal analysis, visualization. **Jian Lei**: methodology, validation, visualization, formal analysis. **Xing‐Hui Zhuang**: methodology, validation, visualization, formal analysis. **Jiang‐Bin Wu**: formal analysis, methodology, validation. **Le‐Le Tang**: methodology, validation, formal analysis. **Hao‐Yu Li**: methodology, validation, formal analysis. **Xiao‐Hui Chen**: methodology, validation, formal analysis. **Yue Wang**: methodology, validation, formal analysis. **Yu‐Mei Li**: conceptualization, funding acquisition, resources, project administration, supervision. **Zhi‐Huang Qiu**: funding acquisition, conceptualization, supervision, resources, project administration. **Zi‐Wen Cai**: conceptualization, funding acquisition, visualization, supervision. **Liang‐Wan Chen**: conceptualization, visualization, funding acquisition, methodology, project administration, resources, supervision.

## Funding

This work was supported by National Natural Science Foundation of China (82570565, 82400300, 82241209), Fujian Provincial Special Reserve Talents Fund (2021‐25), Joint Funds for the Innovation of Science and Technology, Key Laboratory of Cardio‐Thoracic Surgery (Fujian Medical University), Fujian Province University Construction Project (2019‐67), Fujian Provincial Natural Science Foundation of China (2024J08179, 2024J08181), Fujian province (2024Y9271, 2024Y9272, 2025Y9031), and Startup Fund for scientific research, Fujian Medical University (2023QH1047, 2023QH1049).

## Conflicts of Interest

The authors declare no conflicts of interests.

## Supporting information




**Supporting File**: advs78143‐sup‐0001‐SuppMat.docx.

## Data Availability

The data that support the findings of this study are openly available in the NCBI Gene Expression Omnibus (GEO) at https://www.ncbi.nlm.nih.gov/geo/, reference number GSE329036.
