# Supplementary material for: Targeting Nupr1‐Mediated p53 Mitochondrial Sequestration Alleviates Aortic Dissection by Restoring the Gadd45b‐Dependent Survival Program
Source: Adv Sci (Weinh). 2026 Oct 15:e78143. Online ahead of print. doi: 10.1002/advs.78143 (PMC13672869; doi:10.1002/advs.78143)
Supplement: Supplementary file 1 [file ADVS-9999-e78143-s001.docx]

Supporting Information

**Targeting Nupr1-Mediated p53 Mitochondrial Sequestration Alleviates Aortic Dissection by Restoring the Gadd45b-Dependent Survival Program**

*Xi Yang, Ke-Yuan Chen, Yue Tian, Zong-Sheng Jiang, Jian Lei, Xing-Hui Zhuang, Jiang-Bin Wu, Le-Le Tang, Hao-Yu Li, Xiao-Hui Chen, Yue Wang, Yu-Mei Li^*^, Zhi-Huang Qiu^*^, Zi-Wen Cai^*^, Liang-Wan Chen^*^*

**Supplementary Figures**

**Figure S1**

**Figure S1. (A)** through **(F)** Single-cell RNA sequencing result of aortic tissues from patients with non-AD and AD. **(A-F)** UMAP plot analysis of *ACTA2*, *MYH11*, *COL6A1*, *CNN1*, *DCN* and *TAGLN2* between normal and AD groups, respectively. **(G)** Quantification of mean fluorescence intensity of α-SMA and Nupr1 in patients with non-AD and AD groups (*n* = 6 per group). **(H)** Quantification of mean fluorescence intensity of α-SMA and Nupr1 in mice with non-AD and AD groups (*n* = 6 per group).

**Figure S2**

**Figure S2. (A)** Representative images Nupr1 expression using immunofluorescence staining in patients with non-AD and AD aortic sections, and counterstaining with CD31 and DAPI. **(B)** Representative images Nupr1 expression using immunofluorescence staining in patients with non-AD and AD aortic sections, and counterstaining with CD68 and DAPI. **(C)** Quantification of mean fluorescence intensity of CD31 and Nupr1 in mice with non-AD and AD groups (*n* = 6 per group). **(D)** Quantification of mean fluorescence intensity of CD68 and Nupr1 in mice with non-AD and AD groups (*n* = 6 per group).

**Figure S3**

**Figure S3. (A, B)** Blood pressure measurements (*n* = 6 per group) **(C)** Representative images of AAV-sh-Nupr1 transfection efficiency using fluorescence staining in frozen sections, and counterstaining with DAPI. **(D)** The expression of Nupr1 in mice sera from indicated groups was detected by Elisa (*n* = 8 per group). **(E)** Quantification of elastic fiber area (%) of aortic tissues in indicated groups (*n* = 6 per group).

**Figure S4**

**Figure S4. (A)** Representative images of Nupr1 expression using immunofluorescence staining in mice aortic sections, and counterstaining with α-SMA, CD31, PDGFR-α, F4/80 and DAPI, respectively. **(B)** Quantification of Nupr1 mean fluorescence intensity specifically within different regions. Regions of interest were defined by the respective positive signals of markers (e.g., α-SMA for VSMCs, CD31 for endothelia cells, PDGFR-α for adventitial fibroblasts, and F4/80 for macrophages using threshold-based masking in Image J (*n* = 6 per group).

**Figure S5**

**Figure S5. (A)** Expressions and quantification of Nupr1 in VSMCs from indicated groups measured by Western blot (*n* = 4 per group). **(B)** Quantification of nuclear area (*n* = 10 per group). **(C)** Quantification of mitochondrial size (n = 10 per group). **(D)** Representative images of Hsp60 in VMSCs, and counterstaining with DAPI. **(E)** Quantification of relative mitochondrial mass by measuring Hsp60 area per cell (*n* = 6 per group). **(F)** Relative ratio of mtDNA/nDNA in VSMCs under Ang II stimulation (*n* = 6 per group). **(G)** Quantification of fluorescent intensity of Annexin V (*n* = 6 per group).

**Figure S6**

**Figure S6. (A)** RT-qPCR analysis of Nupr1 mRNA levels in VSMCs from Ang II and siRNA-treatment groups. Data were presented as relative fold change to controls (*n* = 3 per group). **(B)** Expressions of Nupr1 from indicated groups measured by Western blot. **(C)** Quantification of ratio of Nupr1 in VSMCs from indicated groups measured by Western blot (*n* = 5 per group). **(D)** Mfn2 and Opa1 of VSMCs from indicated groups measured by Western blot. **(E)** Quantification of expressions of Mfn2 and Opa1 in VSMCs from indicated groups measured by Western blot (*n* = 6 per group). **(F)** Expressions of Bcl2, Bax, cleaved caspase3 and caspase3 of VSMCs from indicated groups measured by Western blot. **(G)** Quantification of expressions of Bax, Bcl2 and ratio of cleaved caspase 3 to caspase 3 in VSMCs from indicated groups measured by Western blot (*n* = 6 per group).

**Figure S7**

**Figure S7. (A)** Cell viability detected in VSMCs treated with Ang II and different concentrations of TFP by CCK8. **(B)** RT-qPCR analysis of Nupr1 mRNA levels in VSMCs from indicated groups. Data were presented as relative fold change to controls (*n* = 3 per group). **(C)** Expressions of Nupr1 in VSMCs from indicated groups measured by Western blot. **(D)** Quantification of Nupr1 expression in VSMCs from indicated groups measured by Western blot (*n* = 6 per group). **(E)** Representative images of fluorescence results showing mPTP opening and Annexin V staining in indicated groups. **(F, G)** Quantification of fluorescence intensity, including Annexin V and calcein (*n* = 6 per group).

**Figure S8**

**Figure S8. (A)** GSEA analysis of p53 pathway. **(B)** Protein-protein interaction analysis of p53 (Trp53) analyzed from STRING database. **(C)** Quantification of nuclear/cytoplasmic fluorescence intensity of ratio of nucleus to cytoplasm (*n* = 10 per group). **D, F, H)** Expressions of p-p53 (Ser20), p-p53 (Ser46) and p53 in cytoplasm, nuclear and mitochondria of VSMCs from indicated groups measured by Western blot, respectively. **E, G, I)** Quantification of p-p53 (Ser20), p-p53 (Ser46), p53, and the ratio of phos-p53(Ser20/Ser46) to p53 in cytoplasm, nuclear and mitochondria of VSMCs from indicated groups measured by Western blot, respectively (*n* = 6 per group). **J, K)** Quantification of p53 expressions and the ratio of phos-p53 (Ser15) to p53 in VSMCs from indicated groups and analyzed by Two-Way ANOVA.

**Figure S9**

**Figure S9. (A)** Real-time monitoring of OCR in VSMCs treated with Gadd45b overexpression detected by O2k. **(B, C, D)** Quantification of mitochondrial maximal respiration, basal respiration and ATP-coupled respiration from indicated groups (*n* = 6 per group). **(E)** Representative images of calcein and Annexin V fluorescence results showing mPTP opening in indicated groups. **(F)** Quantification of fluorescence intensity, including Annexin V and mPTP (*n* = 6 per group). **(G)** Representative images of Rhod-2AM in indicated groups, co-stained with Hoechst 33342 and mito-tracker. **(H)** Quantification of Rhod-2AM fluorescence intensity from indicated groups (*n* = 6 per group). **(I)** Representative images of TUNEL staining in indicated groups and quantification of positive TUNEL staining (*n* = 6 per group).

**Supplementary Tables**

**Table S1**

Comparison of general data between AD patients and non-AD patients

| Parameter | Non-AD (n=18) | AD (n=18) | p |
| --- | --- | --- | --- |
| Age | 59.11±8.281 | 53.44±12.40 | 0.1161 |
| Male/female | 7/11 | 9/9 | 0.7380 |
| BMI (kg/m^2^) | 22.44±4.162 | 27.85±3.076 | **<0.001** |
| Coronary heart disease, n (%) | 0 (0%) | 6 (33.3%) | **0.0191** |
| Hypertension, n (%) | 3 (16.7%) | 10 (55.5%) | **0.0354** |
| Diabetes, n (%) | 2 (11.1%) | 3 (16.7%) | >0.9999 |

**Table S2**

Sense strand sequence

| Target |  | Sequence |
| --- | --- | --- |
| si-Nupr1-1 | Forward (5’→3’) | GGAGAAAUUAGGAGUUGUATT |
|  | Reverse (5’→3’) | UACAACUCCUAAUUUCUCCTT |
| si-Nupr1-2 | Forward (5’→3’) | GGAGAAAUUAGGAGUUGUATT |
|  | Reverse (5’→3’) | UACAACUCCUAAUUUCUCCTT |
| si-Nupr1-3 | Forward (5’→3’) | GGAGAAAUUAGGAGUUGUATT |
|  | Reverse (5’→3’) | UACAACUCCUAAUUUCUCCTT |
| si-Gadd45b-1 | Forward (5’→3’) | UGAAGAGAGCAGAGGCAAUAATT |
|  | Reverse (5’→3’) | UUAUUGCCUCUGCUCUCUUCATT |
| si-Gadd45b-2 | Forward (5’→3’) | CUAUAUCUCUCUAGAGGAATT |
|  | Reverse (5’→3’) | UUCCUCUAGAGAGAUAUAGTT |
| si-Gadd45b-3 | Forward (5’→3’) | UGAUAUCGCUCUGCAGAUUTT |
|  | Reverse (5’→3’) | AAUCUGCAGAGCGAUAUCATT |
| si-NC | Forward (5’→3’) | UUCUCCGAACGUGUCACGUTT |
|  | Reverse (5’→3’) | ACGUGACACGUUCGGAGAATT |

**Table S3**

Antibodies

| Target | Company | | Catalog |
| --- | --- | --- | --- |
| Gapdh | | Beyotime Biotechnology | AF0006 |
| Nupr1 | | Immunoway | YT8077 |
| HRP-labeled Goat Anti-Mouse IgG (H+L) | | Beyotime Biotechnology | A0216 |
| HRP-labeled Goat Anti-Rat IgG (H+L) | | Beyotime Biotechnology | A0208 |
| MFN2 | | Beyotime Biotechnology | AF7473 |
| OPA1 | | Beyotime Biotechnology | AF7653 |
| Myc | | Proteintech | 60003-2-Ig |
| Hsp60 | | Beyotime Biotechnology | AF0186 |
| Gadd45b  phos-p53(Ser 15) | | Invitrogen  Proteintech | PA5-100741  28961-1-AP |
| p53 | | Proteintech | 10442-1-AP |
| Bax  Bcl2  Caspase 3  Cleaved Caspase 3  Lemin B1 | | Beyotime Biotechnology  Beyotime Biotechnology  Beyotime Biotechnology  Beyotime Biotechnology  Beyotime Biotechnology | AF5120  AF6285  ACO30  ACO33  AF5222 |
| α-SMA  F4/80 | | Boster  Abcam | Bm0002  Ab300421 |
| CD31 | | Abcam | Ab9498 |
| PDGFR-α  phos-p53(Ser20)  phos-p53(Ser46) | | Proteintech  Abcam  Proteintech | 84383-2-RR  Ab157454  28960-1-1AP |

**Table S4**

Primer Sequence

| Target |  | | Sequence |
| --- | --- | --- | --- |
| NUPR1-human | | Forward (5’→3’) | GGAAAGGTCGCACCAAGAGAG |
|  | | Reverse (5’→3’) | CTCTCTGAATTCTGCAGCTTGG |
| GAPDH-human | | Forward (5’→3’) | GGTGTGAACCATGAGAAGTATGA |
|  | | Reverse (5’→3’) | GAGTCCTTCCACGATACCAAAG |
| Nupr1-mus | | Forward (5’→3’) | CACCAACAGCCAACCCTTCC |
|  | | Reverse (5’→3’) | CAGCTTCTCTCTTGGTCCGA |
| Gadd45b-mus | | Forward (5’→3’) | ACAACGCGGTTCAGAAGATGC |
|  | | Reverse (5’→3’) | CACATTCATCAGTTTGGCCGC |
| Mdm2-mus | | Forward (5’→3’) | GCAGAAGAAGGCTTGGATGT |
|  | | Reverse (5’→3’) | GATGGTTGGGAATAGTCGTCAC |
| Tuba1b-mus | | Forward (5’→3’) | TGCACTGGTATGTGGGTGAG |
|  | | Reverse (5’→3’) | CTGGAGCAGTTTGACGACAC |
| Caspase9-mus | | Forward (5’→3’) | CCGTGACAAACTTGAGCACC |
|  | | Reverse (5’→3’) | GACCACCACAAAGCAGTCCA |
| Bcl2l11-mus | | Forward (5’→3’) | TGGCCAAGCAACCTTCTGAT |
|  | | Reverse (5’→3’) | GCGGTTCTGTCTGTAGGGAG |
| Dab2ip-mus | | Forward (5’→3’) | GACAACATCATCAGGAAGCAAGT |
|  | | Reverse (5’→3’) | CCAGGCACAGTTCACATAGATAC |
| Gapdh-mus | | Forward (5’→3’) | TGGAAAGCTGTGGCGTGATG |
|  | | Reverse (5’→3’) | TACTTGGCAGGTTTCTCCAGG |
| AAV-Gadd45b | | Forward (5’→3’) | CGACCAAGCCTTGGCTAGCGCGTTTAAACTTAAGCTTGCCAC |
|  | | Reverse (5’→3’) | GTTGATTATCGATAAGCTTGCTGATCAGCGGGTTTAC |
| AAV-sh-Nupr1 | | Forward (5’→3’) | GAGAGAAGCTGCTGCCAATAC |
|  | | Reverse (5’→3’) | GTATTGGCAGCAGCTTCTCTC |
| Target 1 for ChIP | | Forward (5’→3’) | GAATCCCACGCGGGTCTATCT |
|  | | Reverse (5’→3’) | TGAGGCTTCCAAGGGCGTTC |
| Target 2 for ChIP | | Forward (5’→3’) | GACAGCGAGGGGATTCCAGA |
|  | | Reverse (5’→3’) | TCTCCTGATTTCCCGGCAGC |
| Target 3 for ChIP | | Forward (5’→3’) | GAAGCCTCAGTTTCCATTTTGC |
|  | | Reverse (5’→3’) | TGCGTAGCATGAAGCACAAG |
| Target 4 for ChIP | | Forward (5’→3’) | TGCCTCTTGGGTTCGTATCTG |
|  | | Reverse (5’→3’) | CCAGCTCTTCCAGGGTCATG |
| mt-ND1 | | Forward (5’→3’) | CGCCCTAACAACCTATTATCTTCCT |
|  | | Reverse (5’→3’) | CGTGGATAAGATGCTCGGATC |
| mt-CO1 | | Forward (5’→3’) | GTTCTATCAATGGGAGCAGTGTT |
|  | | Reverse (5’→3’) | TCTGAGTAGCGTCGTGGTATT |
| β-actin | | Forward (5’→3’) | AGGGAAATCGTGCGTGAC |
|  | | Reverse (5’→3’) | CATACCCAAGAAGGAAGGCT |

**Uncropped Western Blot data**
